# Methylphenidate Ameliorates Depressive Comorbidity in ADHD Children without any Modification on Differences in Serum Melatonin Concentration between ADHD Subtypes

**DOI:** 10.3390/ijms150917115

**Published:** 2014-09-25

**Authors:** Isabel Cubero-Millán, Antonio Molina-Carballo, Irene Machado-Casas, Luisa Fernández-López, Sylvia Martínez-Serrano, Pilar Tortosa-Pinto, Aida Ruiz-López, Juan-de-Dios Luna-del-Castillo, José Uberos, Antonio Muñoz-Hoyos

**Affiliations:** 1 Neuropediatric, Neuropsicology and Early intervention Unit; Pediatric Service, Clinico San Cecilio Hospital, 18012 Granada, Spain; E-Mails: isacubero83@hotmail.com (I.C.-M.); ireso77@hotmail.com (I.M.-C.); ia_luisita@hotmail.com (L.F.-L.); todosylvia@hotmail.com (S.M.-S.); pilartortosa@yahoo.es (P.T.-P.); aida.ruiz@hotmail.com (A.R.-L.); juberos@ugr.es (J.U.); 2 Biostatistical Department, School of Medicine, Granada University, 18140 Granada, Spain; E-Mail: jdluna@ugr.es

**Keywords:** children, ADHD, ADHD subtypes, comorbidities, depressive symptoms, CDI, prolonged release methylphenidate, melatonin, 6-sulphatoxy-melatonin

## Abstract

The vast majority of Attention-deficit/hyperactivity disorder (ADHD) patients have other associated pathologies, with depressive symptoms as one of the most prevalent. Among the mediators that may participate in ADHD, melatonin is thought to regulate circadian rhythms, neurological function and stress response. To determine (1) the serum baseline daily variations and nocturnal excretion of melatonin in ADHD subtypes and (2) the effect of chronic administration of methylphenidate, as well as the effects on symptomatology, 136 children with ADHD (Diagnostic and Statistical Manual of Mental Disorders, Fourth Edition, Text Revision: DSM-IV-TR criteria) were divided into subgroups using the “Children’s Depression Inventory” (CDI). Blood samples were drawn at 20:00 and 09:00 h, and urine was collected between 21:00 and 09:00 h, at inclusion and after 4.61 ± 2.29 months of treatment. Melatonin and its urine metabolite were measured by radioimmunoassay RIA. Factorial analysis was performed using STATA 12.0. Melatonin was higher predominantly in hyperactive-impulsive/conduct disordered children (PHI/CD) of the ADHD subtype, without the influence of comorbid depressive symptoms. Methylphenidate ameliorated this comorbidity without induction of any changes in the serum melatonin profile, but treatment with it was associated with a decrease in 6-s-melatonin excretion in both ADHD subtypes. Conclusions: In untreated children, partial homeostatic restoration of disrupted neuroendocrine equilibrium most likely led to an increased serum melatonin in PHI/CD children. A differential cerebral melatonin metabolization after methylphenidate may underlie some of the clinical benefit.

## 1. Introduction

Attention-deficit/hyperactivity disorder (ADHD) is the most common neurobehavioral disorder of childhood. In addition to genetic factors [1], environmental risk factors and gender are associated with ADHD [2]. For the vast majority of patients, ADHD is associated with other pathologies, with depressive symptoms as one of the most prevalent [3]. The theories about the neurobiological basis of ADHD have recently centered on two complementary models [4,5], both of which are based on the dysregulation of interacting neural pathways, *i.e*., the inhibitory noradrenergic fronto-cortical activity on dopaminergic striatal structures [6] and the ascending dopamine circuits, in addition to the limbic system [7]. As a neurodevelopmental disorder, in ADHD, there are age-related changes in discrete brain volume areas and connectivity [8] that parallel behavioral improvement and increased efficiency in cognitive task performance [9,10].

Melatonin is a critical circadian synchronizer with a pleiotropic biological signal that exerts multiple effects [11], including increasing tyrosine hydroxylase activity and activating dopamine receptors [12] and sleep/wake cycle regulation [13]. The circadian rhythm of pineal melatonin secretion, which is controlled by the suprachiasmatic nucleus [14], is reflective of the mechanisms that are involved in the control of the sleep/wake cycle. It has been reported that approximately 25% of children with ADHD have some type of sleep disorder, such as delayed sleep phase syndrome [15].

The key features of ADHD include the presence of the core problems of inattention, hyperactivity and impulsivity. In addition, the vast majority of ADHD patients have at least one comorbid condition, e.g., conduct disorders, depressive symptoms or sleep disorders [16]. A hypothetical link between these comorbidities may be the dysregulation of biological rhythms due to alterations in the melatoninergic system [17,18]. In a previous study, both ADHD subtypes had depressive symptom severity equal to a non-ADHD psychiatric control group and greater than community control groups, and externalizing behavior problems and aggression appeared to be related to the hyperactive-impulsive ADHD symptom domain and to overall ADHD symptom severity [19].

The aim of this study was to examine the relationship between serum levels of melatonin, as well as its daily fluctuations in ADHD children in addition to the urinary nocturnal excretion of 6-sulphatoxy-melatonin, prior to and after chronic methylphenidate treatment. In addition, another aim was to explore the relationship of these melatonin values to clinical symptomatology to determine whether this neuroendocrine mediator actively participates in the pathophysiology of ADHD or the response to ADHD treatment.

## 2. Results and Discussion

All of the clinical course data (Evaluation of Deficit of Attention and Hyperactivity (EDAH), d2 attention test and Children’s Depression Inventory (CDI) scores) for the ADHD group, separated for diagnostic subtypes and subgroups, displayed an improvement [20]. After treatment, the increase in height for patients was unaffected, whereas weight decreased, which was expected and previously reported [21]. More than 80% had improvement in parent evaluation data after methylphenidate, with almost 1/3 of participants reporting clinical score data after methylphenidate treatment that no longer meet the ADHD criteria.

At inclusion in the study, 23% of the ADHD sample showed a sleep onset delay, defined by a delay in the hour for going to bed and/or a prolongation of time needed for sleep induction, which were referred to by parents as of low/moderate intensity; with 12% of them showing nocturnal enuresis and no other sleep problems. Approximately a similar percentage (a quarter) of the parents of these subgroups of patients referred to worsening or ameliorations, respectively, of this symptom, with the other 50% of the patients experiencing no changes in their sleep patterns. The amelioration of sleep pattern refers mostly to the decrease of the resistance of children to go to sleep. On the other hand, one of five children without previous sleep disruption referred to a slight increase in the duration time needed to achieve sleep after methylphenidate treatment. Methylphenidate induced no changes in the rate of nocturnal enuresis.

### 2.1. Melatonin Serum Concentration by Attention-Deficit/Hyperactivity Disorder (ADHD) Subtypes and Subgroups

**Figure 1:**
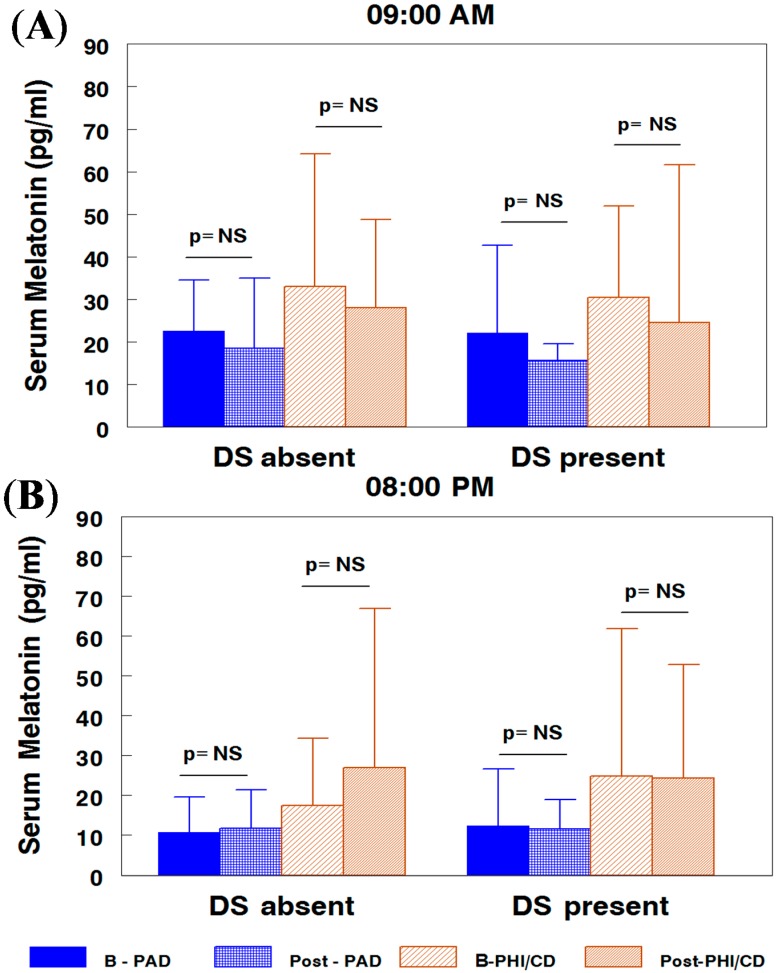
Melatonin concentration in Attention-deficit/hyperactivity disorder (ADHD) children grouped by ADHD subtype and depressive symptoms, in the morning (**A**) and at night (**B**). PAD, predominantly attention disorder; PHI/CD, predominantly hyperactive-impulsive/conduct disordered children.

In the predominantly attention disorder (PAD) ADHD subtype children subgroup without depressive symptoms, the morning melatonin concentration was 22.59 ± 11.97 pg/mL at baseline and 18.58 ± 16.42 pg/mL after treatment (Figure 1A). In children with depressive symptoms, these values were 22.13 ± 20.61 pg/mL before and 15.6 ± 3.99 pg/mL after treatment. At night, the values were slightly lower, 10.7 ± 8.91 and 11.78 ± 9.52 pg/mL, before and after treatment, respectively, in the subgroup without depressive symptoms and (12.35 ± 14.35)/(11.5 ± 7.48) pg/mL, respectively, in the subgroup with depressive symptoms (Figure 1B).

In the hyperactive-impulsive/conduct disordered children (PHI/CD) subtype (Figure 1) subgroup without depressive symptoms, the morning melatonin concentration was 33.11 ± 31.13 and 28.09 ± 20.69 pg/mL after treatment. In children with depressive symptoms, these values were 30.41 ± 21.55 and 24.89 ± 36.98 pg/mL, before and after treatment, respectively. At night, in the subgroup without depressive symptoms, the values were 17.4 ± 16.85 and 27.02 ± 39.9 pg/mL, at baseline and after treatment, respectively, and 24.89 ± 36.98 and 24.36 ± 28.48 pg/mL for subgroup with depressive symptoms, before and after treatment, respectively.

The factorial analysis, adjusted by age and sex, with subtype, subgroup, time and hour, as factors, displayed significant differences between ADHD subtypes with higher values in the PHI/CD children (30.21 ± 27.77 *vs*. 18.62 ± 21.24 pg/mL; *z* = 2.28, *p* = 0.02), with significant day/night fluctuations (*z* = 3.22; *p* < 0.001). There was no differences by time (*z* = 0 and *p* = 0.97) nor depressive symptoms (*z* = 0.1; *p* = 0.94) before or after methylphenidate.

Serum melatonin values were not significantly different in ADHD children *vs.* a control group [21]. Between subtypes and subgroups, we observed a significantly higher PHI/CD than in PAD children, with a similar response to prolonged release methylphenidate (PRMPH) in both subtypes without the influence of comorbid depressive symptoms.

### 2.2. Nocturnal Excretion of 6-Sulphatoxy-melatonin by ADHD Subtypes

**Figure 2:**
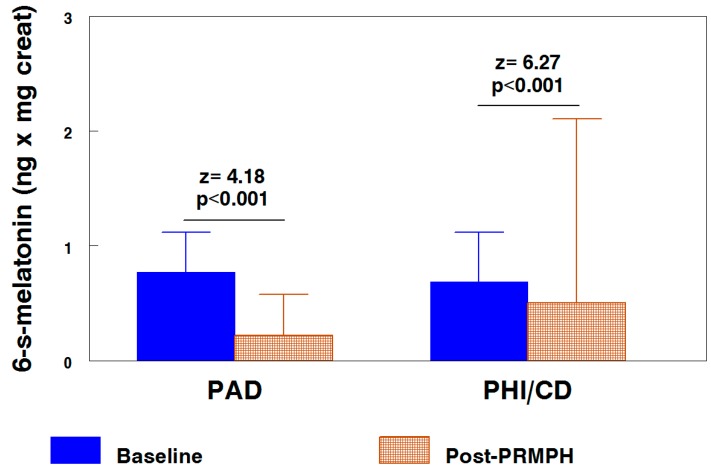
6-Sulphatoxy-melatonin nocturnal excretion, by subtypes and time, in comparisons adjusted by age and sex. PRMPH, prolonged release methylphenidate.

In comparisons adjusted by age and sex, in both ADHD subtypes, PRMPH resulted in a significant decrease in 6-sulphatoxy-melatonin (expressed in ng per mg of creatinine). In the PAD subtype, the values were 0.75 ± 0.34 and 0.24 ± 0.35 before and after treatment (*p* < 0.001), respectively, and 0.72 ± 0.43 and 0.48 ± 1.6 (*p* < 0.001) for the PHI/CD subtype, respectively (Figure 2).

The serum melatonin concentration was significantly greater for the PHI/CD subtype than in the PAD subtype; however, the baseline urinary excretion of 6-sulphatoxy-melatonin (adjusted comparison) was very similar in both subtypes. The treatment with PRMPH induced a very significant decrease in excretion of 6-*S*-melatonin in both subtypes (Figure 2), which was even greater for the PAD subtype according to visual inspection of the figure, although the value of the *z* is lower because, in this subtype, the “*n*” of the sample was much lower.

In another report, we demonstrated that neurosteroids and other neuroendocrine mediators are also influenced by PRMPH treatment. Dehydroepiandrosterone and allopregnanolone displays a trend toward lower baseline concentrations in ADHD children [20]. Methylphenidate exerts a differential effect on their concentration as a function of depressive symptoms, because, *i.e*., PRMPH induced a very significant increase in the concentration of allopregnanolone, only in PAD patients without comorbid depressive symptoms.

In the melatonin case, on the contrary, this paper demonstrates that the occurrence of depressive symptoms does not modify melatonin concentration, in either of the ADHD subtypes, nor at baseline, nor after PRMPH treatment. Baseline melatonin was significantly higher in the PHI-CD ADHD subtype *vs*. the PAD-ADHD subtype. The PHI-CD subtype is more related with externalizing symptoms and higher melatonin levels that of the PAD-ADHD subtype, which is more related to internalizing symptoms. In a control group without ADHD, the serum melatonin levels concentration were intermediate between PAD and PHI/CD subtypes, without differences in comparisons with both ADHD subtypes [21]. PRMPH ameliorates clinical depressive symptoms, as reflected by the decrease of CDI total punctuation for both subgroups PAD: (12.44 ± 7.30)/(11.11 ± 6.12) (*p* = 0.054), PHI/CD: (13.44 ± 6.24)/(12.33 ± 7.80) (*p* < 0.001), before/after treatment, respectively (Table 1), although with significance differences in the PHI/CD subgroup, most likely due to the higher “*n*” sample number.

Our data reinforce the need to quantify ADHD comorbidities in clinical practice settings, as this can help to better define the profile of the patient and, thus, adapt the treatment protocol to the patient’s needs and to reformulate family expectations.

**Table 1:** Children’s Depression Inventory (CDI) values, by ADHD subtypes and subgroups, before and after prolonged release of methylphenidate treatment.

Total CDI	Time	PAD (*n* = 37)		PHI/CD (*n* = 99)		Total (*n* = 136)	*z*	*p*
<18 (*n* = 104)	Baseline Post-PRMPH	10.18 ± 4.74		10.48 ± 4.21		10.39 ± 4.34	−1.743	0.081
		9.47 ± 5.08		10.97 ± 6.34		10.45 ± 5.93		
		9.76 ± 4.49		10.65 ± 5.05				
		*z*	*p*	*z*	*p*			
		−2.09	0.037	−0.84	0.40			
>17 (*n* = 32)	Baseline Post-PRMPH	22.40 ± 8.73		21.53 ± 2.59		21.71 ± 4.31	−3.301	0.001
		20.50 ± 8.96		14.86 ± 4.29		16.11 ± 5.84		
		22.00 ± 6.99		18.70 ± 4.74				
		*z*	*p*	*z*	*p*			
		−0.921	0.357	−3.30	0.001			
Total Simple (*n* = 136)	Baseline Post-PRMPH	12.44 ± 7.30		13.44 ± 6.24		13.16 ± 6.52	−3.596	0.0001
		11.11 ± 6.12		12.33 ± 7.80		12.25 ± 6.19		
		11.89 ± 6.80		12.97 ± 6.06				
		*z*	*p*	*z*	*p*			
		−1.93	0.054	−3.48	0.001			

Related samples: Wilcoxon signed rank test, two-sided test. The values are expressed as the mean ± SD.

Our data indicate that fewer ADHD symptoms (EDAH scale) and fewer depressive symptoms (CDI) after methylphenidate may be related, in part, to the neuroendocrine changes documented in this study. In addition, subtle changes in the daily fluctuations of both melatonin and serotonin [21] may contribute to marked clinical improvement in the key symptoms of ADHD. Melatonin and serotonin influence food intake [22], immunity [23], neurological function [24] and stress response [25]. In addition to its effects on sleep regulation, its salivary levels have been correlated with ADHD psychopathology [26], and melatonin treatment could exert some neuroprotective effects [27].

The serum melatonin values of the PAD subtype are more correlated with the decrease observed in other mediators, for example for adrenocorticotropic Hormone (ACTH), β-endorphin and melatonin in affective deprived children [25] and for children with other types of stress [28]. Although neuroendocrine mediator increases in response to stress are adaptive in the short term, animal models of chronic stress and depression indicated lower brain and plasma concentrations of several mediators in response to acute stressors. These results are consistent with our results in children [20,25]. Two neurosteroids [29], dehydroepiandrosterone and allopregnanolone, displayed slightly lower values (without significant differences) compared with a control group. The baseline concentrations and responses to PRMPH differ for both neurosteroids. In the case of allopregnanolone, the presence of comorbid depressive symptoms erase the very high increase of concentration after methylphenidate that is observed in the PAD subtype without these comorbidities. On the contrary, dehydroepiandrosterone displayed slightly higher values in the subgroup of PHI/CD-ADHD with depressive symptoms and a further increase after PRMPH. Melatonin [30] and the neurosteroid, dehydroepiandrosterone [31], share antiglucocorticoid properties that may have regulatory effects on glucocorticoid action in the brain [32].

Untreated children with ADHD have a high rate of sleep onset disorder [33], which may be associated with combined-type ADHD, which may reflect the association between sleep problems and more severe ADHD symptoms [34]. Defiant behavior at bedtime causes delays in getting into bed and falling asleep and reinforces the need for using sleep medication [35]. The stimulant medication for ADHD may aggravate sleep onset delay [16], and melatonin has been demonstrated to be an effective therapy in the long term for the treatment of chronic sleep onset insomnia in children with ADHD [36]. As combined-type ADHD children may more frequently need to use melatonin, the melatonin increase that our report demonstrated in this subtype may reflect an incomplete restoration of their physiological needs. Other mechanisms may help explain that PAD-ADHD children displayed even lower melatonin concentrations and suffer from less intense sleep onset delays.

The reduced excretion of the 6-sulphatoxy-melatonin in nocturnal collected urine after PRMPH prompt us to suggest that the stimulant treatment may induce an alternative route for melatonin metabolism/utilization. Until the discovery of two 5-methoxylated kynuramines, named *N*(1)-acetyl-*N*(2)-formyl-5-methoxykynuramine (AFMK) and *N*(1)-acetyl-5-methoxy-kynuramine (AMK), melatonin was usually believed to be almost exclusively metabolized to 6-hydroxymelatonin and its excretion product, 6-sulphatoxy-melatonin. AFMK and AMK now are known as major brain metabolites of melatonin [37], with activities as potent cyclooxygenase inhibitors, NO scavengers, inhibitor and/or downregulators of neuronal and inducible NO synthases and mitochondrial metabolism modulators [38]. These properties may underline some of the beneficial effects of methylphenidate.

Psychostimulants, such as methylphenidate, produce differential lasting behavioral alterations, depending on the time of the day that they are administered, and correlate with diurnal changes in the system of transcription factors, termed clock genes, and with changes in the availability of subtypes of dopamine receptors [39]. The molecular mechanism of melatonin’s effects on the responsiveness of CNS to psychostimulants appears to involve melatonin receptors and clock genes. In addition to their benefits, psychostimulants also produce toxic effects in the brain [40,41] that are believed to be due to oxidative stress in addition to the stimulant-induced depletion of striatal dopamine [42]. *In vitro* studies have demonstrated that amphetamine increases inducible NOS mRNA, which may be prevented by melatonin [39,43].

The PAD and PHI-CD ADHD subtypes may be separate disorders. Attention and impulsivity are sexually dimorphic in healthy populations. These gender differences may be related to dehydroepiandrosterone [44]. Similar to our data, experimental [45] and clinical studies [46] have reported significant inverse correlations between clinical symptomatology (in particular hyperactivity symptomatology) and dehydroepiandrosterone levels [47,48]. Moreover, symptoms of hyperactivity and impulsivity in attention-deficit hyperactivity disorder may be separately regulated at the level of the nucleus accumbens [49].

Melatonin production, which is related to free radical production [50], reduces the production of adhesion molecules and pro-inflammatory cytokines [51], has antiapoptotic activity [52] and functions as a direct and indirect antioxidant, scavenging free radicals, stimulating antioxidant enzymes and enhancing the activities of other antioxidants or protecting other antioxidant enzymes from oxidative damage [53,54]. Melatonin has also been demonstrated to stimulate neurogenesis [55]. Melatonin could contribute to the prevention of environmental risk factors by gender that are associated with ADHD [2] and with other disorders [56] that may be related to oxidative stress [57]. Moreover, some of the deleterious effects associated with the highly effective use of psychostimulants in ADHD may be prevented by melatonin [39,43]. We now hypothesize that nocturnal administration of melatonin may be helpful for treatment of both ADHD subtypes.

In terms of the limitations of our study, our study had an open design and lack of randomization, with reporting of objective neuroendocrine measures of response after chronic treatment. Other limitations include (1) a low number of females, adolescents and patients belonging to the PAD subtype and (2) a large proportion of ADHD children with comorbid CD. Similar studies involving homogeneous groups of patients in terms of age, gender and co-morbidities, along with a more precise estimation of the adherence to treatment, are warranted for defining the serum biomarkers of the disorder and its comorbidities, in addition to the neurophysiological biomarkers that recently have been proposed [58].

## 3. Experimental Section

### 3.1. Sample

A total of 148 children (115 males, 33 females) between the ages of 5 and 14 years old (mean: 9.61 ± 2.54 year) were included in a prospective, quasi-experimental open clinical study in a hospital-based sample, primarily reporting objective neuroendocrine measures of response.

The sample included a total of 136 children who met the Diagnostic and Statistical Manual of Mental Disorders, Fourth Edition, Text Revision/9th International Classification of Diseases (DSM-IV-TR/ICD-9) criteria for ADHD [59], after completing the clinical protocol to exclude the main comorbidities, in which each included patient was assessed at least twice, before and after treatment. Consequently, each patient may be considered as his/her own control.

### 3.2. Clinical Method

After the initial clinical interview with parents, completion of a personal medical history and physical examination of the child, we delivered to parents the following documents: (1) the DSM-IV-TR criteria assessment, which was completed by the child’s teacher; (2) EDAH scale (Spanish acronym for Evaluation of Deficit of Attention and Hyperactivity scale [60,61]), in duplicate, one for the teacher and the other for the child’s parents; (3) the CDI, which was completed by subjects aged ≥8 years; and (4) a sleep diary that was completed for one week. The EDAH contains some of the main criteria recommended in the DSM-IV-TR to aid in identifying children with ADHD and conduct disorder (CD). The EDAH questionnaire is a 20-item scale [62] that utilizes structured observation by teachers and is divided into two 10-item subscales for ADHD and CD. Based on EDAH, the ADHD group was sub-classified into two clinical subtypes: children with predominantly attention deficit (PAD; if AD (attention deficit) > 9; HI (hyperactivity-impulsivity) < 10; and total scores < 30) and children with predominantly hyperactive-impulsive/conduct disorder (PHI/CD; if AD < 10; H (hyperactivity) > 9; and/or total punctuation > 29). Therefore, of the 78 children who were included in the PHI/CD group, 34 of them (44%) met criteria for the diagnosis of HI without CD. Of the 44 children with symptoms of CD, 33 displayed a predominance of symptoms of HI on the symptoms of CD, whereas the rest of the children (11/78; 14%) had a prevalence of symptoms of CD on the symptoms of HI. Only 26 of 78 children in this group (33%) did not meet further criteria for attention deficit.

The d2 attention test [63] is a measure of attention, particularly visual attention. d2 measures processing speed, rule compliance and quality of performance, allowing for a neuropsychological estimation of individual attention and concentration performance, by quantification of two scoring keys: errors of omission and errors of commission. The d2 test has been fully validated and includes extensive norms according to age, sex and education.

The CDI [64] is a self-report assessment of depression for children whose two subscales (negative mood and negative self-esteem) consist of the items that are most unique to depression and least related to anxiety. For defining subgroups, we considered the sum of both subscales, with a cut-off of >17 points considered pathological. The depressive symptom was assessed through interviews with the parents at baseline, in the clinical follow-up and quantified by the CDI score fulfilled by each children.

All children were evaluated with an abbreviated intelligence test as a screening cognitive ability Kaufman (KBIT) [65] and also completed the Spanish version of the Sleep Diary of the National Sleep Foundation for one week, and the ADHD group completed the diary once again after treatment.

Written informed consent was obtained from all parents and from children aged ≥12 years, and informed assent was obtained from all participants. The study design and outcome variables were approved by the Hospital Ethics Committee and the Health Research Fund of Spanish Ministry of Science and Innovation.

The exclusion criteria were as follows: (1) KBIT < 85; (2) preexisting or actual treatment for epilepsy; (3) other treatments for ADHD or other conditions and (4) revocation of previous informed consent.

Table 1 shows the clinical characteristics of the two study subgroups at inclusion. Methylphenidate (Osmotic Release Oral System (OROS) formulation) was well tolerated.

Table 2 shows the incidence of depressive symptoms separated by ADHD subtype and sex.

**Table 2:** Sample distribution of ADHD subtypes by presence of depressive symptoms and sex. The values are expressed as number and percentage.

Sex	Depressive Symptoms	ADHD Subtype		Total (%)
		PAD	PHI/CD	
Boys	No	24 (77.42)	61 (80.26)	85 (79.43)
	Yes	7 (22.58)	15 (19.74)	22 (20.56)
	Total	31 (28.97)	76 (71.03)	107 (78.67)
Girls	No	5 (83.33)	14 (60.87)	19 (65.52)
	Yes	1 (16.67)	9 (39.13)	10 (34.48)
	Total	6 (20.69)	23 (79.31)	29 (21.33)
Total	No	29 (78.38)	75 (75.75)	104 (76.47)
	Yes	8 (21.62)	24 (24.24)	32 (23.53)
	Total	37 (21.01)	99 (72.99)	136 (100)

#### 3.2.1. Treatment

The only drug used in the study was prolonged release methylphenidate (PRMPH, OROS formulation), initially at 0.5 mg/kg/day. The dosage was adjusted as a function of response and tolerance to treatment (absence of adverse symptomatology). The mean initial dose of methylphenidate was 25.81 ± 10.35 mg, and the final dose at the time of the second evaluation was 31.85 ± 10.68 mg. At inclusion, all patients were naive of any medication, and no other treatment (pharmacological or psychological) was administered before conclusion of the protocol.

Previously, at inclusion and during the study duration time, none of the patients of our sample were treated with melatonin or other sleep medications.

#### 3.2.2. Measurements

None of the samples were obtained in the presence of an acute or severe illness. Blood samples were taken at 20:00 and at 09:00 the following day. In the ADHD group, after 4.61 ± 2.29 months of daily methylphenidate administered early in the morning, the identical study protocol was repeated. Serum was separated into 0.5-mL aliquots for freezing at −30 °C until analysis.

### 3.3. Analytical Method

Serum melatonin was measured using melatonin direct RIA (IBL–Hamburg, Germany). The intra- and inter-assay CV were 3.9%–6.9% in the range of 28.8 to 266 pg/mL and 6.2%–15.9% in the range of 3.5 to 281 pg/mL, respectively. The mean recovery of melatonin was 102%, and the sensitivity was 0.9 pg/mL.

In addition, 6-sulphatoxy-melatonin (6-S-aMT) in urine was measured by ELISA (IBL–Hamburg). The detection limit was 1 ng/mL, with an intra-assay range of 5.2% to 12.2%, and the inter-assay range was 5.1%–14.9%. Recovery ranged from 91% to 122%, and the correlation with RIA techniques was *r* = 0.96.

### 3.4. Statistical Method

To achieve the objectives of the study, factorial analyses were conducted as described below. For comparisons between EDAH and CDI scores (ordinal variables), Wilcoxon signed-rank tests (paired samples) were used for inferential statistics. For comparisons between patients (cases) and each variable in the study, the factors in the factorial models were as follows: (1) subtype with two categories: PAD and PHI/CD subtypes; (2) patients, nested in subtypes and subgroups (CDI); (3) hour, with two categories, day and night, and crossed with subtype; and (4) time, with two levels before and after treatment; this factor was a crossed factor with subtype and hour. Subtype, hour and time were fixed effects factors, and patients were considered as a random effects factor. Comparisons between cases were performed. The factorial model had the following three factors: (1) group with two categories (PAD and PHI/CD subtypes); (2) patients nested in CDI subgroups; and (3) hour, with two categories, day and night, that was crossed with group. Group and hour were fixed effects factors, and subjects was a random effects factor. For both types of comparisons, an ANOVA table was built, and higher interactions were determined. If these were significant, multiple pairwise comparisons were made using Bonferroni’s correction, and if not, these corrections were applied to the principal effects in the table. The experimental quantities for these comparisons were not “*t*” as expected, because we used “*z*”, the normal approximations for “*t*’s”, because of global sample sizes. The analyses reported were crude analyses, and adjusted analyses by age and gender were performed using the ANCOVA methodology. In all cases, the interactions were studied for levels below 0.15, and the latest comparisons were considered significant at *p* < 0.05 after applying the penalty provided by the correction. When analyzing the variances in different groups, homogeneous transformations were conducted for data using natural logarithm to achieve uniformity. We used the statistical package STATA 12.0 (StataCorp, College Station, TX, USA) for all analyses.

## 4. Conclusions

In summary, our study indicates that the presence of depressive symptoms is not responsible for the observed higher melatonin levels in the PHI-CD subtype of ADHD children, although the pineal hormone may participate in the pathophysiology of ADHD, as, in addition to melatonin alleviating sleep onset disorders, clinically effective methylphenidate treatment is related to a decrease of 6-sulphatoxy-melatonin excretion, most likely indicating a differential cerebral metabolism, which may generate end products that finally result in a clinically favorable outcome. Methylphenidate appears to induce changes in several others neuroendocrine mediators that globally act by adjusting physiological functions that collaborate to achieve the high efficacy of the stimulant pharmacological treatment of ADHD.
